# Supplementary material for: Resolving the full spectrum of human genome variation using Linked-Reads
Source: Genome Res. 2019 Apr;29(4):635–45. doi: 10.1101/gr.234443.118 (PMC6442396; doi:10.1101/gr.234443.118)
Supplement: Supplemental Material [file supp_gr.234443.118_Supplemental_longranger-2.2.zip › longranger-2.2.tar.bz2!/lr2.2-public/lib/stan/cmdstan/stan/doc/api/html/functions_e.html]

Stan: Class Members


|  |  |
| --- | --- |
|  | Stan  2.5.0  probability, sampling & optimization |


- Main Page
- Related Pages
- Namespaces
- Classes
- Files

- Class List
- Class Index
- Class Hierarchy
- Class Members

- All
- Functions
- Variables
- Typedefs
- Enumerator
- Related Functions

- \_
- a
- b
- c
- d
- e
- f
- g
- h
- i
- j
- k
- l
- m
- n
- o
- p
- q
- r
- s
- t
- u
- v
- w
- x
- y
- z
- ~


All Classes Namespaces Files Functions Variables Typedefs Enumerations Enumerator Friends Macros Pages

Here is a list of all class members with links to the classes they belong to:

### - e -

- effective\_sample\_size()
  : stan::mcmc::chains< RNG >
- end\_adaptation\_window()
  : stan::mcmc::windowed\_adaptation
- end\_array()
  : stan::json::json\_data\_handler
  , stan::json::json\_handler
- end\_object()
  : stan::json::json\_data\_handler
  , stan::json::json\_handler
- end\_text()
  : stan::json::json\_data\_handler
  , stan::json::json\_handler
- end\_update\_p()
  : stan::mcmc::base\_leapfrog< H, P >
  , stan::mcmc::expl\_leapfrog< H, P >
- engage\_adaptation()
  : stan::mcmc::base\_adapter
- engine
  : stan::io::stan\_csv\_metadata
- epsilon()
  : Eigen::NumTraits< stan::agrad::var >
  , std::numeric\_limits< stan::agrad::fvar< T > >
  , std::numeric\_limits< stan::agrad::var >
- epsilon\_
  : stan::mcmc::base\_hmc< M, P, H, I, BaseRNG >
- epsilon\_jitter\_
  : stan::mcmc::base\_hmc< M, P, H, I, BaseRNG >
- err\_stream\_
  : stan::mcmc::base\_hamiltonian< M, P, BaseRNG >
  , stan::mcmc::base\_mcmc
- error\_msgs\_
  : data\_only\_expression
  , stan::gm::bare\_type\_grammar< Iterator >
  , stan::gm::expression07\_grammar< Iterator >
  , stan::gm::expression\_grammar< Iterator >
  , stan::gm::functions\_grammar< Iterator >
  , stan::gm::program\_grammar< Iterator >
  , stan::gm::returns\_type\_vis
  , stan::gm::statement\_2\_grammar< Iterator >
  , stan::gm::statement\_grammar< Iterator >
  , stan::gm::term\_grammar< Iterator >
  , stan::gm::var\_decls\_grammar< Iterator >
- estimator\_
  : stan::mcmc::covar\_adaptation
  , stan::mcmc::var\_adaptation
- estimator\_name\_
  : stan::mcmc::windowed\_adaptation
- evolve()
  : stan::mcmc::base\_integrator< H, P >
  , stan::mcmc::base\_leapfrog< H, P >
- exists()
  : stan::gm::variable\_map
- expl\_leapfrog()
  : stan::mcmc::expl\_leapfrog< H, P >
- exponentiated\_factor\_r
  : stan::gm::term\_grammar< Iterator >
- expr\_
  : stan::gm::assignment
  , stan::gm::expression
  , stan::gm::index\_op
  , stan::gm::sample
- expr\_type()
  : stan::gm::expr\_type
- expression()
  : stan::gm::expression
- expression07\_g
  : stan::gm::expression\_grammar< Iterator >
  , stan::gm::var\_decls\_grammar< Iterator >
- expression07\_grammar()
  : stan::gm::expression07\_grammar< Iterator >
- expression07\_r
  : stan::gm::expression07\_grammar< Iterator >
- expression09\_r
  : stan::gm::expression\_grammar< Iterator >
- expression10\_r
  : stan::gm::expression\_grammar< Iterator >
- expression14\_r
  : stan::gm::expression\_grammar< Iterator >
- expression\_g
  : stan::gm::program\_grammar< Iterator >
  , stan::gm::statement\_2\_grammar< Iterator >
  , stan::gm::statement\_grammar< Iterator >
  , stan::gm::term\_grammar< Iterator >
  , stan::gm::var\_decls\_grammar< Iterator >
- expression\_grammar()
  : stan::gm::expression\_grammar< Iterator >
- expression\_r
  : stan::gm::expression\_grammar< Iterator >
- expression\_t
  : stan::gm::expression
- expression\_type()
  : stan::gm::expression
- expression\_visgen()
  : stan::gm::expression\_visgen


---

[ Stan Home Page ]

*© 2011–2014,
Stan Development Team.*
